# *Ehrlichia* spp*.* infection in rural dogs from remote indigenous villages in north-eastern Brazil

**DOI:** 10.1186/s13071-018-2738-3

**Published:** 2018-03-20

**Authors:** Filipe Dantas-Torres, Yury Yzabella da Silva, Débora Elienai de Oliveira Miranda, Kamila Gaudêncio da Silva Sales, Luciana Aguiar Figueredo, Domenico Otranto

**Affiliations:** 1 Department of Immunology, Aggeu Magalhães Institute, Oswaldo Cruz Foundation (Fiocruz), Recife, Brazil; 2 Department of Veterinary Medicine, University of Bari, Valenzano, Italy

**Keywords:** *Ehrlichia canis*, Ticks, *Rhipicephalus sanguineus* (*sensu lato*), Brazil

## Abstract

**Background:**

*Ehrlichia canis* is a tick-borne bacterium that causes severe, life-threatening disease in dogs, being more prevalent in tropical and subtropical countries. Randomized studies conducted in Brazil indicate that the prevalence of *E. canis* infection in dogs ranges from 0.7% to over 50.0%. In a study conducted in northern Brazil, the prevalence was higher in dogs from urban areas, as compared to dogs from rural areas. In the present study, we investigated the exposure to *Ehrlichia* spp. infection in dogs from remote indigenous villages located in a rural area in north-eastern Brazil.

**Methods:**

From March to June 2015, 300 privately owned dogs were blood sampled and tested by a rapid ELISA and by a conventional PCR in order to detect anti-*Ehrlichia* spp. antibodies and *E. canis* DNA, respectively. Additionally, dogs were also tested for anti-*Anaplasma* spp. antibodies and *Anaplasma platys* DNA, using the same diagnostic approaches. Positivity was correlated with tick infestation and dogs’ data (gender, age and level of restriction).

**Results:**

Overall, 212 (70.7%) dogs were positive for at least one test targeting *Ehrlichia* spp. In particular, 173 (57.7%) dogs were positive only by rapid ELISA, 5 (1.7%) only by PCR and 34 (11.4%) were simultaneously positive by both tests. In the same way, 39 (13.0%) dogs presented detectable *E. canis* DNA in their blood, whereas 18 (6.0%) dogs were *A. platys* DNA-positive. Coupling serological and PCR data, 63 (21.0%) dogs were simultaneously positive to *Ehrlichia* spp. and *Anaplasma* spp. Positivity rates for both *Ehrlichia* spp. and *Anaplasma* spp. were higher among dogs more than 1 year of age. Sick dogs were more positive to *Ehrlichia* spp. as compared to healthy dogs.

**Conclusions:**

Dogs from rural areas in north-eastern Brazil are highly exposed to *Ehrlichia* spp. infection and positivity rates do not necessarily correlate with current tick infestation load, since only one infected tick bite is needed to get the infection. This reinforces the importance of keeping dogs free of ticks, in order to reduce as much as possible the risk of infection by *E. canis* and other tick-borne pathogens such as *Babesia vogeli*, which are usually co-endemic.

## Background

Canine monocytic ehrlichiosis is a life-threatening tick-borne disease characterized by nonspecific clinical signs, such as fever, weakness, lethargy, anorexia, lymphadenomegaly, splenomegaly, hepatomegaly and weight loss [1]. The disease is caused by the intracellular bacterium *Ehrlichia canis*, which affects dogs worldwide, being more prevalent in tropical and subtropical regions, where the brown dog tick *Rhipicephalus sanguineus* (*sensu lato*), the primary tick vector, is abundant [2]. For instance, prevalence rates of *E. canis* infection in dogs range from less than 1% up to 50% in Europe and it is higher in kennelled dogs and in dogs without external antiparasitic treatment [1]. Indeed, *E. canis* is endemic in all European countries bordering the Mediterranean Sea [1], where the tick vectors are highly abundant, particularly from spring to autumn [3].

The prevalence of *E. canis* infection in dogs varies according to several factors, but generally correlates with the level of exposure to infected tick vectors. Studies have reported higher positivity rates among males as compared to females and among older dogs as compared with young ones [1]. This is probably related to behavioural characteristics of males, which increase their level of exposure to the tick vectors in comparison with females. The same applies for age since the probability of becoming infected increases as the dog ages. Breed-related susceptibility has also been suggested by epidemiological and experimental studies. Indeed, German shepherd dogs and Siberian Huskies are predisposed to developing more severe clinical signs [4, 5].

Canine monocytic ehrlichiosis is a very common disease in Brazil, where dogs are highly exposed to brown dog ticks [6, 7]. Randomized studies conducted in several regions of Brazil indicate that the prevalence of *E. canis* infection in dogs ranges from 0.7% to over 50.0% [7–10]. A study conducted in Roraima, northern Brazil, reported that the prevalence of *E. canis* infection was higher in dogs from urban areas, as compared to dogs from rural areas [11], whereas a more recent study conducted in Paraná, southern Brazil, indicated the opposite [12].

Recently, we reported a high level (58%) of exposure to *Ehrlichia* spp*.* among privately owned dogs living in Goiana [6], a city situated in the north-east region of Pernambuco State, north-eastern Brazil. In the present study, we investigated the exposure to *Ehrlichia* spp. infection in rural dogs from remote indigenous villages in the countryside of Pernambuco State.

## Methods

### Study area and sample size calculation

The present randomised prevalence study was conducted in four indigenous villages in the municipality of Pesqueira (08°21'42"S, 36°41'41"W; 654 m above sea level), Pernambuco State. Members of the tribe Xukuru de Ororubá inhabit these villages, which are located in the scrub zone of Pernambuco, 204 km far from Recife, the state’s capital. Semi-restricted or unrestricted dogs are frequent in these villages and are commonly used as guard or as pets. The climate is semi-arid, hot and dry, with annual average temperature of 23 °C and average precipitation of 700 mm, with rains concentrated from February to July.

For this study, the minimum sample size (*n* = 243) was calculated considering a margin of error of 5%, a confidence level of 95%, a population size of 20,000 (unknown), and an expected prevalence of 20.0%, based on the average prevalence found in a large study conducted in Brazil [13].

### Physical examination and blood sampling

From March to June 2015, a total of 300 privately owned dogs were visited by a veterinarian and by a field team in previously selected indigenous villages. Each dog was physically examined for clinical signs suggestive of vector-borne diseases, including weight loss, pale mucous membranes, enlarged lymph nodes and petechiae.

The number of ticks and fleas was estimated by the attending veterinarian, during the physical examination, through visual inspection. The level of infestation was classified as negative (no ticks or fleas), low (1–3 ticks and 1–5 fleas), moderate (4–10 ticks and 6–20 fleas) and high (> 10 ticks and > 20 fleas) [14]. Ticks, fleas and also lice were manually collected and preserved in labelled vial containing 70% ethanol for later morphological identification [15, 16].

Dogs were physically restrained by their owners and blood samples (~5 ml) were withdrawn from their cephalic, jugular or femoral veins. An aliquot (~2 ml) was placed in an EDTA tube (Vacuette® K3E K3EDTA tube, Greiner Bio-One GmbH, Kremsmünster, Austria) and other (~3 ml) in a serum separator tube (Vacuette® Z Serum Separator Clot Activator tube, Greiner Bio-One GmbH). Samples were maintained on ice until processing. In the laboratory, EDTA-treated blood samples were frozen at -20 °C until DNA extraction using PureLink® Genomic DNA Mini Kit (Invitrogen, Carlsbad, USA), according to the manufacturer’s instructions. Serum separator tubes were centrifuged at 2,000× *g* for 10 min and obtained serum samples were frozen at -20 °C until serological testing.

### Diagnostic testing

Serum samples were tested by a rapid ELISA (SNAP® 4Dx Plus Test, IDEXX Laboratories, Maine, USA), which detects antibodies to *Anaplasma* spp. (*A. platys*/*A. phagocytophilum*), *Ehrlichia* spp. (*E. canis*/*E. ewingii*), *Borrelia burgdorferi*, and antigens of *Dirofilaria immitis*. All tests were performed according to the manufacturer’s instructions.

*Ehrlichia canis* DNA was detected by conventional PCR amplifying a 410 bp fragment of the heat shock protein (*groEL*) gene using the species-specific primers gro-E.canis163s and groEcanis573as [17]. Each reaction mixture contained 7.5 μl of DNA-free water, 1.5 μl of each primer at a concentration of 10 pmol/μl, 12.5 μl GoTaq® Colorless Master Mix (Promega, Madison, USA) and 2 μl of the sample DNA to be tested, totalling 25 μl. Thermal cycling conditions were as follows: initial denaturation at 95 °C for 30 s, followed by 40 cycles of 94 °C for 10 s, 62 °C for 15 s and 72 °C for 15 s, with a final extension of 72 °C for 1 min.

*Anaplasma platys* DNA was detected by conventional PCR amplifying a 515 bp region of the *groEL* gene using the species-specific primers GroAplatys-35s and GroAplatys-550as [18]. Each reaction contained 7.5 μl of DNA-free water, 12.5 μl of GoTaq® Colorless Master Mix (Promega), 1.5 μl of each primer at a concentration of 10 pmol/μl and 2 μl of DNA sample, totalling 25 μl. Thermal cycling conditions were as follows: initial denaturation at 95 °C for 1 min, followed by 55 cycles of 94 °C for 15 s, 62 °C for 15 s and 72 °C for 15 s.

DNA extracted from naturally infected dogs (with *E. canis* or *A. platys*) was used as positive control and DNA-free water as negative control. PCR products were separated by electrophoresis in a 1.5% agarose gel, stained with ethidium bromide, and visualised by UV transillumination.

### Data analysis

The 95% confidence intervals (95% CI) of positivity rates were calculated. The Chi-square (*χ*^2^) or G-test was used to compare positivity rates relative to sex (male *vs* female), age (≤ 1 year *vs* > 1 year), clinical status (healthy, sick), level of tick/flea infestation (absent *vs* low *vs* moderate *vs* high), and level of restriction (restricted *vs* semi-restricted). The differences were considered statistically significant when *P* ≤ 0.05. Statistical analysis was performed using BioEstat, version 5.3 [19].

## Results

All 300 dogs included in the present study were mongrels, of which 179 (59.7%) were males and 121 (40.4%) were females. The great majority of the dogs were > 1 year old (77.4%) and presented at least one clinical sign suggestive of vector-borne diseases (59.7%). Thirty-five (11.7%) dogs were considered restricted and 265 (88.4%) semi-restricted.

Ticks were detected in 91 (30.4%; 95% CI: 25.1–35.5%) dogs, of which 19 (20.9%) presented high, 23 (25.3%) medium and 49 (53.9%) low levels of infestation. Most ticks collected (97.3%) were identified as *R. sanguineus* (*s.l.*) (157 males, 100 females and 28 nymphs). Four dogs were also infested by *Amblyomma parvum* (5 females) and 2 by *Rhipicephalus microplus* (3 females). By comparing tick infestation levels and positivity rates to both *Ehrlichia* spp. (*χ*^2^ = 3.235, *df* = 3, *P* = 0.3568) and *Anaplasma* spp. (*G* = 2.3918, *df* = 1, *P* = 0.4952), no significant differences were found. Fleas were observed in 133 (44.4%; 95% CI: 38.7–50.0%) dogs, of which 29 (21.8%) presented high, 33 (24.9%) medium and 71 (53.4%) low levels of infestation. All fleas collected (40 males and 73 females) were identified as *Ctenocephalides felis felis*. By comparing flea infestation levels and positivity rates to *Ehrlichia* spp. (*χ*^2^ = 10.099, *df* = 3, *P* = 0.0177), a significant difference was found. Indeed, the highest positivity rate to *Ehrlichia* spp. (76.1%) was recorded among flea-free dogs. No significant difference was found in relation to *Anaplasma* spp. positivity and flea infestation (*χ*^2^ = 1.544, *df* = 3, *P* = 0.6722).

In addition to ticks and fleas, eight dogs were infested by lice, which were all identified as *Heterodoxus spiniger* (7 males, 14 females and 4 nymphs).

Overall, 212 (70. 7%; 95% CI: 65.5–75.8%) dogs were positive for at least one test targeting *Ehrlichia* spp. In particular, 173 (57.7%) dogs were positive only by rapid ELISA, 5 (1.7%) only by PCR and 34 (11.4%) were simultaneously positive by both tests. *Anaplasma* spp. infection was detected in 72 (24.0%; 95% CI: 19.2–28.8%) dogs, of which 54 (18.0%) were positive only by rapid ELISA, 11 (3.7%) only by PCR and 7 (2.4%) were simultaneously positive by both tests. Sixty-three (21.0%) dogs were simultaneously positive to *Ehrlichia* spp. and *Anaplasma* spp. Positivity rates for both *Ehrlichia* spp. (*χ*^2^ = 40.662, *df* = 1, *P* = 0.0001) and *Anaplasma* spp. (*χ*^2^ = 4.164, *df* = 1, *P* = 0.0413) were higher among dogs more than 1 year of age. Sick dogs were more exposed to *Ehrlichia* spp. (*χ*^2^ = 6.039, *df* = 1, *P* = 0.0140) as compared to healthy dogs. Statistical data on comparisons made between different variables and positivity rates to both *Ehrlichia* spp. and *Anaplasma* spp. are summarised in Table 1.

**Table 1:** Comparisons between positivity rates to *Ehrlichia* spp. and *Anaplasma* spp. and different variables

Variables		*n*	*Ehrlichia* spp.			*Anaplasma* spp.		
			Positive	% (95% CI)	Statistical analysis	Positive	% (95% CI)	Statistical analysis
Age	< 1	68	27	39.7 (28.1–51.3)	*χ*^2^ = 40.662, *df* = 1, *P* = 0.0001	10	14.7 (6.3–23.1)	*χ*^2^ = 4.164, *df* = 1, *P* = 0.0413
	≥ 1	232	185	79.8 (74.4–84.9)		62	26.8 (21–32.4)	
Gender	Male	179	132	73.8 (67.3–80.2)	*χ*^2^ = 2.026, *df* = 1, *P* = 0.1546	40	22.4 (16.2–28.4)	*χ*^2^ = 0.665, *df* = 1, *P* = 0.4147
	Female	121	80	66.2 (57.7–74.5)		32	26.5 (18.6–34.3)	
Tick infestation	Absent	209	150	71.8 (65.7–77.9)	*χ*^2^ = 3.235, *df* = 3, *P* = 0.3568	48	23.0 (17.3–28.7)	*G* = 2.3918, *df* = 1, *P* = 0.4952
	Low	49	35	71.5 (58.8–84.1)		10	20.4 (9.1–31.7)	
	Medium	23	17	73.9 (not calculated)		8	34.8 (15.3–54.2)	
	High	19	10	52.6 (not calculated)		6	31.6 (not calculated)	
Flea infestation	Absent	167	127	76.1 (69.6–82.5)	*χ*^2^ = 10.099, *df* = 3, *P* = 0.0177	40	24.0 (17.5–30.4)	*χ*^2^ = 1.544, *df* = 3, *P* = 0.6722
	Low	71	47	66.2 (55.2–77.2)		20	28.2 (17.7–38.6)	
	Medium	33	24	72.8 (57.5–87.9)		7	21.3 (7.3–35.2)	
	High	29	14	48.3 (30.1–66.5)		5	17.2 (not calculated)	
Clinical status	Sick	179	136	76.0 (69.7–82.2)	*χ*^2^ = 6.039, *df* = 1, *P* = 0.0140	46	25.7 (19.3–32.1)	*χ*^2^ = 0.702, *df* = 1, *P* = 0.4022
	Healthy	121	76	62.8 (54.2–71.4)		26	21.5 (14.2–28.8)	
Level of restriction	Restricted	35	23	65.8 (50.0–81.4)	*χ*^2^ = 0.469, *df* = 1, *P* = 0.4935	6	17.1 (not calculated)	*χ*^2^ = 1.021, *df* = 1, *P* = 0.3122
	Semi-restricted	265	189	71.4 (65.9–76.8)		66	24.9 (19.7–30.1)	

## Discussion

Our results indicate that dogs from rural areas in north-eastern Brazil are highly exposed to *Ehrlichia* spp. infection, in spite of the relatively low prevalence of tick infestation (30.33%) found in the studied population. Considering that most rural dogs are semi-restricted or unrestricted and usually untreated against ticks, the low prevalence of tick infestation was unexpected. Indeed, in other studies conducted in Pernambuco, the prevalence of tick infestation ranged from 41.7% [6] to 58.5% [20] in urban and rural dogs, respectively. As an example, a recent study carried out in south-western Pernambuco, indicated that rural dogs were generally more infested by both ticks and fleas, as compared with urban dogs [21] with a tick infestation rate ranging from 44.4% to 50.8% in urban and rural dogs, respectively. Overall, this is in agreement with previous studies conducted in Pernambuco [6, 20]. However, we should keep in mind that, besides environmental conditions (e.g. rural *versus* urban landscapes), the level of ectoparasite infestation in dogs is also related to other factors, including the owner’s capability to afford preventive measures [6] and therefore, the risk of tick infestation might be extremely high in urban dogs as well. In a study conducted in the Metropolitan region of Recife, tick infestation rates reached 79.3% in owned dogs attended at a public veterinary clinic and 93.3% in stray dogs [22]. It is worth mentioning that stray dogs may act as reservoirs of many kinds of parasites, especially in low-income countries [23].

Considering the low percentage of tick-infested dogs, one would expect a low level of exposure to tick-borne pathogens. Unexpectedly, we found a high positivity rate (70.67%) to *Ehrlichia* spp. Most randomized studies carried out in Brazil suggest that the prevalence of *E. canis* infection in dogs hardly ever surpasses 50.0% [7], whereas in non-randomized studies on dogs presenting suggestive clinical signs of canine monocytic ehrlichiosis, it may reach over 90.0% (e.g. [24]). In our study, the positivity rate among dogs displaying suggestive clinical signs was 76.0%, being significantly higher (*χ*^2^ = 6.039, *df* = 1, *P* = 0.0140) than that detected in healthy dogs (62.8%). This is relevant also considering that “prevalence studies” conducted with dogs attended at veterinary hospitals or clinics, might be biased, overestimating the actual prevalence of *E. canis* infection.

Two studies comparing the positivity rates to *E. canis* in urban *versus* rural dogs reported that urban ones were significantly more exposed to the infection [11, 12]. Altogether, these findings suggest that both rural and urban dogs might be highly exposed to *E. canis* infection, and that the risk of infection is not necessarily linked to the level of tick infestation. Indeed, in theory, only one infected tick feeding for some hours is sufficient for transmission to occur.

For quite some time, it has been acknowledged that the longer the tick blood-feeding period, the higher the risk of pathogen transmission. Pioneer studies conducted at the dawn of the 1900s indicated that ticks usually required a ten hour feeding period to transmit *Rickettsia rickettsii* (the causative agent of Rocky Mountain spotted fever) to vertebrate hosts [25, 26]. However, ticks that had previously fed on another host (interrupted feeding) required a shorter period (minimum of one hour and 45 minutes) to transmit the bacterium or even less, as recently demonstrated [27]. In recent decades, our knowledge on the transmission times of several tick-borne pathogens has increased considerably [28]. For instance, it has been ascertained that *E. canis* requires a minimum period of three hours to be transmitted by *R. sanguineus* (*s.l.*) to a susceptible vertebrate host [29]. This information is of practical significance, particularly when planning prevention strategies against *E. canis*, using repellent, fast killing products.

## Conclusions

Altogether, our results indicate that dogs from rural areas in north-eastern Brazil are highly exposed to *Ehrlichia* spp. infection and that positivity rates do not necessarily correlate with tick infestation load; that is to say, only one infected tick bite is needed to get a dog infected. This reinforces the importance of keeping dogs free of ticks, in order to reduce as much as possible to risk of infection by *E. canis* and other tick-borne pathogens such as *Babesia vogeli*, which are usually co-endemic.

## Abbreviations

EDTA: ethylenediamine tetraacetic acid
DNA: deoxyribonucleic acid
ELISA: enzyme-linked immunosorbent assay
PCR: polymerase chain reaction
UV: ultraviolet
CI: confidence interval
*s.l.*: *sensu lato*
*df*: degrees of freedom
